# Structural analysis of $\hbox{Sn}$ on ${\hbox{Au}(111)}$ at low coverages: Towards the ${\hbox {Au}_{2}\hbox {Sn}}$ surface alloy with alternating fcc and hcp domains

**DOI:** 10.1038/s41598-025-91733-2

**Published:** 2025-03-07

**Authors:** Julian A. Hochhaus, Stefanie Hilgers, Marie Schmitz, Lukas Kesper, Ulf Berges, Carsten Westphal

**Affiliations:** 1 Department of Physics, TU Dortmund University, Otto-Hahn-Str. 4a, 44227 Dortmund, Germany; 2 DELTA, Center for Synchrotron Radiation, TU Dortmund University, 44227 Dortmund, Germany

**Keywords:** Two-dimensional materials, Surfaces, interfaces and thin films

## Abstract

We report on the structural and chemical evolution of submonolayer $\hbox{Sn}$ on ${\hbox{Au}(111)}$ up to the formation of the striped ${\hbox {Au}_{2}\hbox {Sn}}$ surface alloy. Using Low-Energy Electron Diffraction (LEED) and Scanning Tunneling Microscopy (STM), we identify a previously unobserved hexagonal $(2\times 2)$-reconstruction at a $\hbox{Sn}$ film thickness of $\approx 0.28$ monolayers (ML). X-ray Photoelectron Spectroscopy (XPS) analysis reveals that the $(2\times 2)$-structure is not chemically bonded to the ${\hbox{Au}(111)}$ substrate. With increasing $\hbox{Sn}$ coverage, the $(2\times 2)$-reconstruction performs a structural transition into a mixed phase before forming a local $(\sqrt{3} \times \sqrt{3})\text {R}{30}^{\circ }$-reconstruction at a $\hbox{Sn}$ film thickness of $0.33\,\textrm{ML}$. This reconstruction is superimposed by a larger periodicity resembling the herringbone reconstruction of clean ${\hbox{Au}(111)}$. Our XPS analysis identifies this phase as an ${\hbox {Au}_{2}\hbox {Sn}}$-alloy. By combining high-resolution x-ray photoelectron diffraction (XPD) measurements of $\hbox{Au}\,\hbox{4f}$ and $\hbox{Sn}\,\hbox{4d}$ 4d core levels with simulations based on a genetic algorithm, we propose a structural model for the ${\hbox {Au}_{2}\hbox {Sn}}$-supercell, revealing an unusually large unit cell with $\text {Rec}(26\times \sqrt{3})$-periodicity. This study advances the understanding of the structural evolution of $\hbox{Sn}$ surface reconstructions on ${\hbox{Au}(111)}$ up to the formation of the ${\hbox {Au}_{2}\hbox {Sn}}$ surface alloy. Furthermore, it provides insights into the structural arrangements emerging at higher submonolayer $\hbox{Sn}$ coverages on ${\hbox{Au}(111)}$, offering potential pathways towards realizing freestanding stanene.

## Introduction

Since the discovery of graphene, the exploration of two-dimensional materials has undergone remarkable advancements. Within the carbon group, 2D counterparts such as silicene^1^, germanene^2^, stanene^3^, and plumbene^4 ^have attracted significant attention, particularly for their unique electronic properties, including linear Dirac-type band dispersion^5^. The heavier elements in this group are especially intriguing due to their enhanced spin-orbit coupling, which introduces topological characteristics that position these materials as promising candidates for next-generation electronic applications^6^. Among them, stanene, the honeycomb arrangement of $\hbox{Sn}$ atoms, has generated significant interest due to predictions from theory of its quantum spin Hall insulating phases, a bandgap of up to ${300\,\hbox{meV}}$^7,8^, and topological superconductivity^9^. These properties hold promise for novel room-temperature applications of topological phenomena.

Stanene was first experimentally synthesized on ${\hbox {Bi}_{2}\hbox {Te}_3}$^3^. Later investigations of stanene on ${\hbox{InSb}(111)}$ revealed an unusually large bandgap of ${440\,\hbox{meV}}$, exceeding theoretical predictions^10^. Metal substrates have also been investigated as platforms for supporting epitaxial stanene growth. These include the formation of ultra-flat, planar stanene on ${\hbox{Cu}(111)}$^11^ and the ${\hbox {Ag}_{2}\hbox {Sn}}$-alloy formed on ${\hbox{Au}(111)}$^12^, as well as buckled stanene grown on ${\hbox{Sb}(111)}$^13^. Theoretical calculations predict that ${\hbox{Au}(111)}$ is a promising candidate for supporting planar stanene growth^14^. Experimentally, it has been found that the growth of $\hbox{Sn}$ on $\hbox {Au}(111)$ results in numerous distinct structural arrangements. However, the existing literature presents conflicting results regarding the structural arrangement of the adsorbed $\hbox{Sn}$ atoms and the chemical interaction at the $\hbox{Sn}$-$\hbox{Au}$ interface.

Starting with a local $(\sqrt{3}\times \sqrt{3})\text {R}{30}^{\circ }$-arrangement at $0.33\,\textrm{ML}$ coverage, an ${\hbox {Au}_{2}\hbox {Sn}}$-alloy with linear band dispersion was reported as the first periodic arrangement observed at low coverages. However, different lattice parameters of the superimposed structure have been published in the literature^15,16^. Several structural arrangements were reported for an increased film thickness of $0.66\,\textrm{ML}$, depending on the post-deposition annealing temperature. These include an incommensurable phase which is either claimed to be a mix of a striped-like and honeycomb arrangement of $\hbox{Sn}$ atoms^17^, or an $\hbox{AuSn}$ alloy phase^18 ^with parabolic electron-like dispersion bands^19^. At elevated temperatures, a $(\sqrt{3}\times \sqrt{7})$-arrangement was reported to be either a stretched $\hbox{Sn}$ honeycomb arrangement with linear disperging bands and high Fermi velocity^20^ or an ${\hbox {Au}_{2}\hbox {Sn}}$-alloy^19^. Finally, at even further raised annealing temperatures, an ${\hbox {Au}_{2}\hbox {Sn}}$-alloy was reported^15,19^.

Since the structure arrangement of $\hbox{Sn}$ on ${\hbox{Au}(111)}$ is still unclear, we investigated the structural evolution of $\hbox{Sn}$ on ${\hbox{Au}(111)}$ at low film thicknesses of $\le 0.33\,\textrm{ML}$. To our knowledge, no or only vague structural information is available in the literature under this coverage regime. In this study, we present the first report on the structural evolution of periodic $\hbox{Sn}$-arrangements leading to the formation of the ${\hbox {Au}_{2}\hbox {Sn}}$-alloy at $0.33\,\textrm{ML}$. Using LEED, STM, and XPS, we identify the formation of a previously unobserved hexagonal $(2\times 2)$-reconstruction at ${\approx 0.28\,\textrm{ML}}$ $\hbox{Sn}$ coverage. This phase transitions through a mixed phase, which appears as a striped-like pattern in STM, into a $(\sqrt{3}\times \sqrt{3})\text {R}{30}{^{\circ }}$-reconstructed ${\hbox {Au}_{2}\hbox {Sn}}$-alloy phase. A detailed XPS analysis reveals the chemical evolution during this process and confirms that the $(2\times 2)$ is not strongly bonded to the Au surface. This phase might serve as a precursor to a honeycomb arrangement of $\hbox{Sn}$ on ${\hbox{Au}(111)}$, as reported at higher coverages^17^.

The ongoing discussion in the literature regarding the supercell of the $(\sqrt{3}\times \sqrt{3})\text {R}{30}{^{\circ }}$ ${\hbox {Au}_{2}\hbox {Sn}}$-alloy^15,16^ is addressed using XPD measurements. Combined with simulations using a genetic algorithm, we reveal an unusually large unit cell with $\text {Rec}(26\times \sqrt{3})$-periodicity. Our insights on the ${\hbox {Au}_{2}\hbox {Sn}}$-alloy help to enable the growth of strain-free honeycomb stanene, which was predicted by theory on ${\hbox {Au}_{2}\hbox {Sn}}$^21 ^and already experimentally demonstrated on ${\hbox {Pd}_{2}\hbox {Sn}}$^12^.

## Results and discussion

**Fig. 1:**
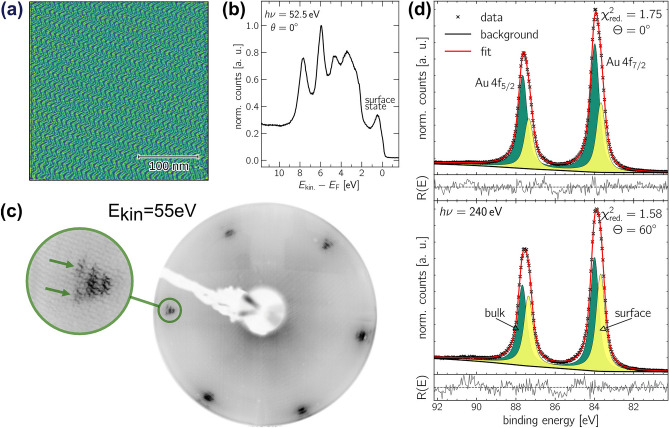
Clean ${\hbox{Au}(111)}$ surface in well-ordered herringbone reconstruction. **(a)** Large area (${{300}\times {300}\,\hbox{nm}^{2}}$) constant-current STM image measured at $U ={-1.3}\,\hbox {V}$ and $I ={23}\,\hbox {pA}$. **(b)** Valence band spectrum recorded at a photon energy of $\hbox{h}\nu ={52.5}\,\hbox {eV}$ under normal emission. **(c)** The LEED pattern was obtained with $E_{\text {kin.}}={55}\,\hbox {eV}$. In the inset, the superstructure spots of the herringbone reconstruction are marked by arrows. **(d)** High-resolution XPS spectra of the $\hbox{Au}\,\hbox{4f}$ signal, recorded at a photon energy of $\hbox{h}\nu ={240}\,\hbox {eV}$ and under emission angles of $\Theta ={0}^{\circ}$ (top) and $\Theta ={60}^{\circ }$ (bottom).

The preparation of the clean ${\hbox{Au}(111)}$ surface was verified using XPS to confirm chemical purity and LEED in combination with STM to assess long-range order, identifying the well-known herringbone reconstruction of the clean ${\hbox{Au}(111)}$ surface. After performing multiple cycles of sputtering and annealing, the results for the prepared ${\hbox{Au}(111)}$ crystal are shown in Figure 1.

Figure 1(a) displays an STM image of the characteristic herringbone reconstruction of ${\hbox{Au}(111)}$, with terraces showing an undistorted herringbone pattern over widths exceeding ${400\,\hbox{nm}}$. The valence band spectrum in Figure 1(b) reveals the Shockley surface state at $\approx {480}\,\hbox {meV}$ below the Fermi edge, which matches to literature^22^and confirms a well-prepared, clean surface^23^. The long-range order of the ${\hbox{Au}(111)}$ surface was checked by LEED. As a result, a perfect herringbone reconstruction was obtained, as seconded by the LEED picture presented in Figure 1(c). The set of sharp satellite spots around the first-order diffraction spots, marked by green arrows in the inset, provides clear evidence of the herringbone reconstruction.

Figure 1(d) presents angle-resolved XPS spectra of the $\hbox{Au}\,\hbox{4f}$ core level. The binding energy scale was calibrated to the Fermi edge for each measurement. Two distinct components were identified: the bulk component, plotted in green at $E_{\text {bin}} = {84.00}\,\hbox {eV}$, and the surface component, plotted in yellow, which becomes more pronounced at the more surface-sensitive emission angle of $\Theta = {60}^{\circ }$. The surface component attributed to the herringbone reconstruction is shifted by ${\approx {0.3}\,\hbox {eV}}$ to lower binding energies. A small asymmetry of $\beta = 0.03$ was observed in the $\hbox{Au}\,\hbox{4f}$ signals, linked to the electronic structure of noble metals^24,25^. The corresponding fit parameters are shown in Table 1. The obtained binding energy $E_{\text {bin}}$ and asymmetry parameter $\beta$ are in excellent agreement with the literature^26–29^. The high-resolution spectra did not detect any component indicating possible contamination, which is discussed in detail in the XPS section below.

**Table 1:** Fit parameters of the XPS analysis corresponding to the $\hbox{Au}\,\hbox{4f}$ core-level signal in Figure 1(d).

Structural phase	$\Theta$ ($^{\circ }$)	Component	$E_{\text {bin}}$ ($\hbox {eV}$)	$E_{\text {SOC}}$ ($\hbox {eV}$)	FWHM ($\hbox {eV}$)	asymmetry $\beta$	rel. area ($\%$)
clean $\hbox{Au}$ subfigure 1(d)	0	bulk	84.01	3.67	0.53	0.03	64.83
		surface	83.70	3.67	0.53	0.03	35.17
	60	bulk	84.00	3.67	0.53	0.03	53.90
		surface	83.69	3.67	0.53	0.03	46.10

### LEED

**Fig. 2:**
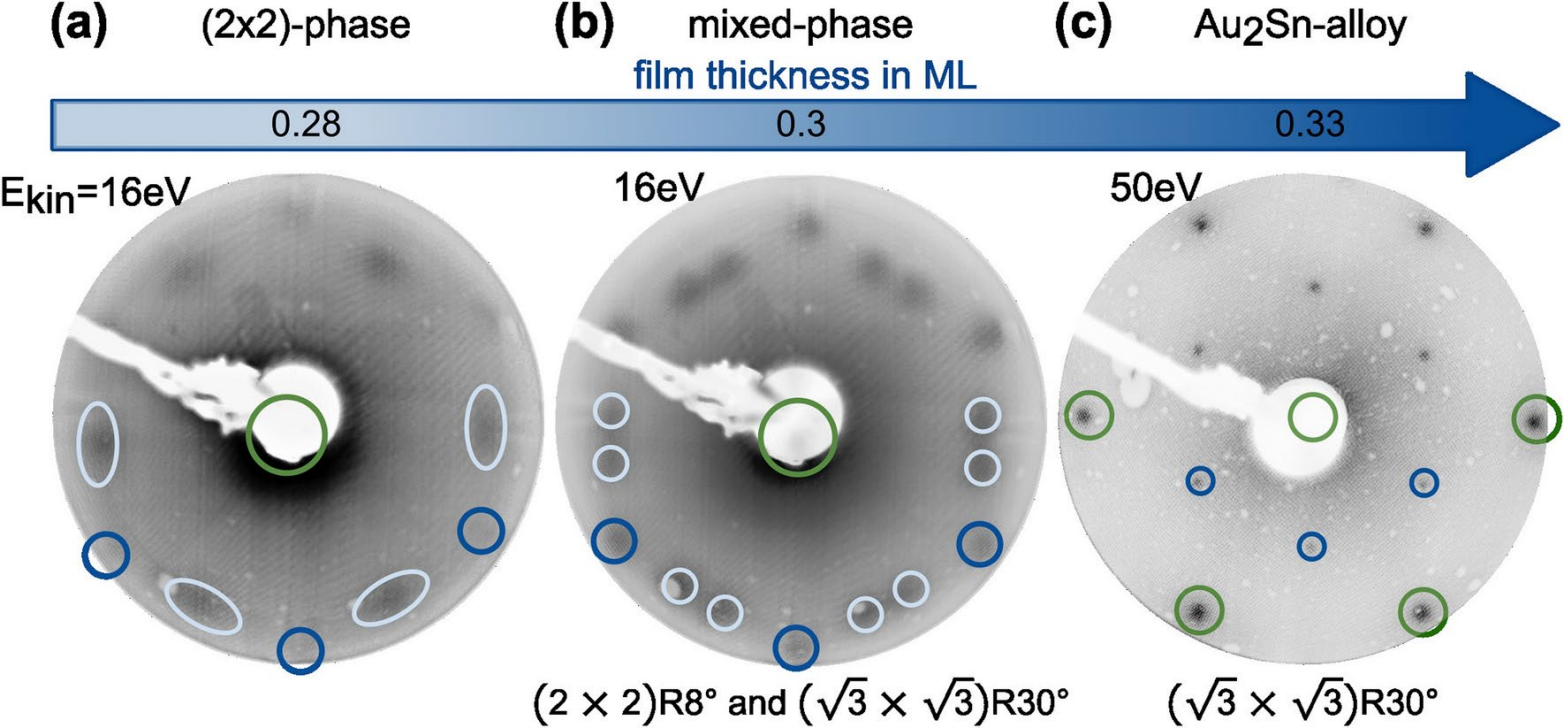
Overview of the structural evolution at different coverages depicted by LEED patterns. The LEEDPat simulations identify the reflection spots of different structural phases: dark green circles correspond to the $\hbox{Au}$ lattice. Meanwhile, dark and light blue circles indicate $\hbox{Sn}$ spots of different domains.

After preparing the clean $\hbox{{Au}(111)}$ surface, submonolayer films of tin were deposited in small increments while maintaining the substrate at room temperature. The $\hbox{Sn}$-film thickness, expressed in monolayers ($\hbox{ML}$), is defined with respect to the substrate crystal as detailed in the Methods section. The structural arrangement of $\hbox{Sn}$ was examined at each deposition step using LEED. Slight variations in film thickness yielded distinct structures, as shown in Figure 2. The periodicity of each structure was measured relative to the $\hbox{Au}$ substrate spots and subsequently simulated by using LEEDPat^30^. Simulated lattice patterns, indicated by dark and light blue circles in Figure 2, represent the diffraction pattern of the $\hbox{Sn}$ film, while green markers correspond to substrate reflexes.

For coverages $\le 0.25\,\textrm{ML}$, no periodic arrangement of $\hbox{Sn}$ was detected by LEED, while the herringbone reconstruction remained visible. At $\approx 0.25\,\textrm{ML}$, a faint $(2\times 2)$-reconstruction appears, intensifying at $\approx 0.28\,\textrm{ML}$ before fading again at higher coverages. Light blue ovals mark the $(2\times 2)$-reflexes. No second diffraction order was observed; only the first-order diffraction spots were visible at low kinetic energies ($E_{\text {kin.}}\le {25}\,\hbox {eV}$). This indicates that the $(2\times 2)$-structure is not well-ordered on a long range. Additionally, faint diffraction spots from a $(\sqrt{3}\times \sqrt{3})\text {R}{30}^{\circ }$-phase appear between the $(2\times 2)$-spots, as indicated by dark blue circles in Figure 2(a).

As the coverage increases to $\approx 0.3\,\textrm{ML}$, the $(\sqrt{3}\times \sqrt{3})\text {R}{30}^{\circ }$ reflexes become more prominent, as shown in Figure 2(b). Notably, the previously blurred $(2\times 2)$-spots observed at lower coverages transform into two sharper spots near the $(2\times 2)$-position, corresponding to a $(2\times 2)\text {R}{8}^{\circ }$-reconstruction. This transition indicates that the emergence of the $(\sqrt{3}\times \sqrt{3})\text {R}{30}^{\circ }$-phase enhances the ordering of the $(2\times 2)$-structure. Additionally, the presence of second- and third-order diffraction spots from the $(\sqrt{3}\times \sqrt{3})\text {R}{30}^{\circ }$-phase confirms long-range ordering. In contrast, still only the first-order diffraction spots of the $(2\times 2)\text {R}{8}^{\circ }$-structure remain visible at low kinetic energies.

With a further increase in film thickness to approximately $0.33\,\textrm{ML}$ the $(\sqrt{3}\times \sqrt{3})\text {R}{30}^{\circ }$-phase remains visible with now very sharp reflexes, as displayed in Figure 2(c). This type of reconstruction has been observed for various materials deposited on noble metal crystals with a (111)-orientation, where one out of three atoms of the (111)-surface is substituted, leading to a surface alloy. Examples include ${\hbox {Cu}_{2}\hbox {Bi}}$^31,32^, ${\hbox {Cu}_{2}\hbox {Sb}}$^33^, ${\hbox {Ag}_{2}\hbox {Ge}}$^34–36^, ${\hbox {Ag}_{2}\hbox {Sb}}$^33^, ${\hbox {Ag}_{2}\hbox {Pb}}$^37,38^, ${\hbox {Pd}_{2}\hbox {Sn}}$^39^, and ${\hbox {Ag}_{2}\hbox {Sn}}$^40,41^. Moreover, it has been found that these surface alloys may play an important role in the growth of strain-free single atomic 2D layers, as predicted for ${\hbox {Au}_{2}\hbox {Sn}}$^21^ and demonstrated for stanene on ${\hbox {Pd}_{2}\hbox {Sn}}$^12^.

The clean $\hbox{Au(111)}$ surface differs from other noble metal substrates due to its complex $\text {Rec}(22\times \sqrt{3})$ herringbone reconstruction^42^. Despite this, several alloy phases of tin on ${\hbox{Au}(111)}$ have been observed, such as ${\hbox {Au}_{2}\hbox {Sn}}$ in a $(\sqrt{3}\times \sqrt{7})$-arrangement^19^, $\hbox{AuSn}$ ${\hbox{p}(3\times 3)\text {R}{15}^{\circ }}$^18,19^, and $\hbox{Au}_{5.1}\hbox{Sn}$ in a $(\sqrt{3}\times \sqrt{3})\text {R}{30}^{\circ }$-configuration^43^. Additionally, the ${\hbox {Au}_{2}\hbox {Sn}}$-alloy has been reported in the $(\sqrt{3}\times \sqrt{3})\text {R}{30}^{\circ }$-arrangement by Maniraj et al^15^. and Shah et al^16^..

While Maniraj et al. suggested that the herringbone reconstruction remains unchanged during the formation of the ${\hbox {Au}_{2}\hbox {Sn}}$-alloy, Shah et al. identified a striped-like order in STM images, reminiscent of the herringbone pattern but with a larger unit cell of $\text {Rec}(26\times \sqrt{3})$.

### XPS

**Fig. 3:**
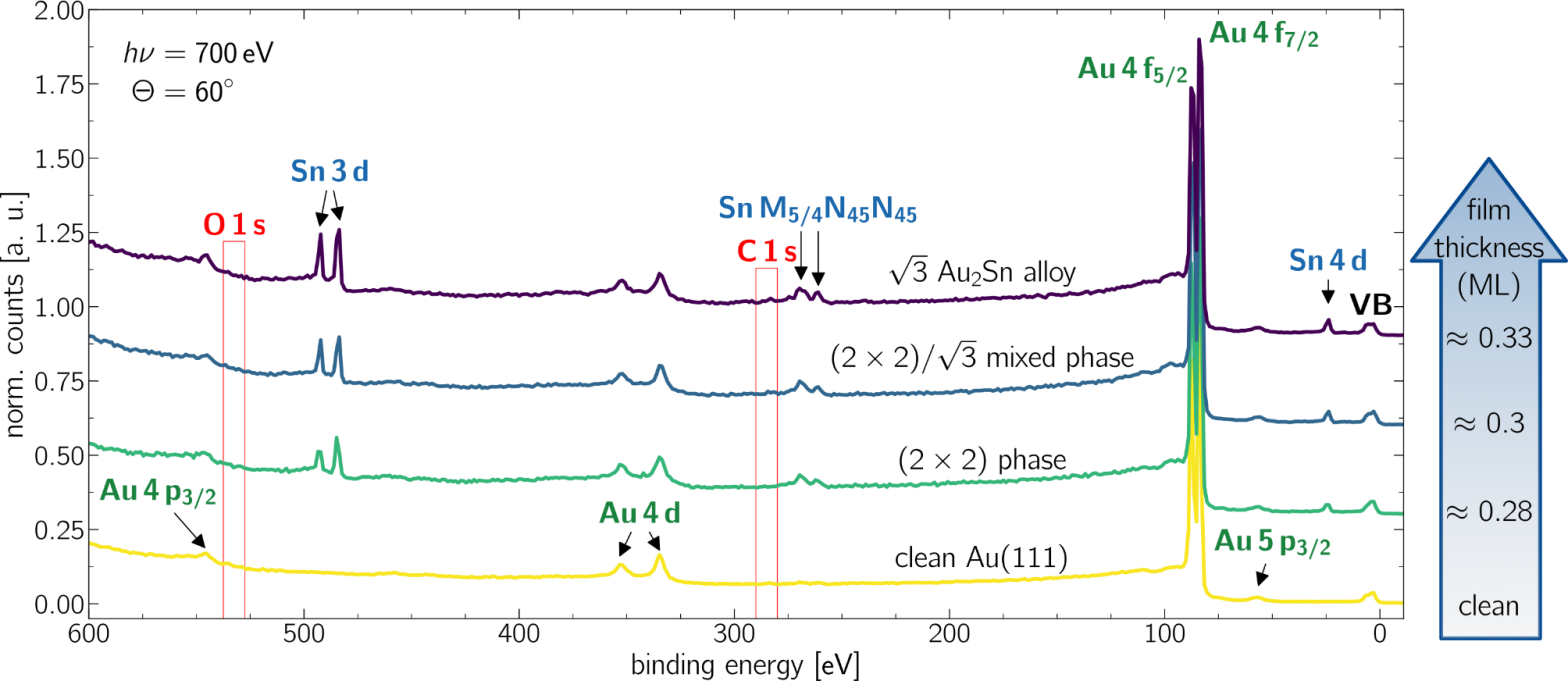
XPS survey spectra. The data were obtained for the clean $\hbox{{Au}(111)}$ sample and the low-coverage $\hbox{Sn}$-phases. The measurements were taken at $\hbox{h}\nu ={700}\,\hbox {eV}$ and at $\Theta ={60}^{\circ }$ emission angle. The binding energy of the spectra was referenced to the Fermi edge.

XPS survey spectra were recorded with an excitation energy of $\hbox{h}\nu ={700}\,\hbox {eV}$ to get an overview of the chemical composition of all observed structural phases. Figure 3 displays the respective spectra taken at an emission angle of $\Theta ={60}^{\circ }$ for enhanced surface sensitivity. The spectrum obtained for the clean $\hbox{{Au}(111)}$ substrate is shown at the bottom of Figure 3, while spectra recorded for increasing $\hbox{Sn}$ submonolayer film-thicknesses are stacked above. No residual contamination was detected; the expected signals of common contaminants such as $\hbox{C}\,\hbox{1s}$ and $\hbox{O}\,\hbox{1s}$ were not found, as indicated by red boxes in Figure 3. The increasing thickness of the $\hbox{Sn}$ film is shown by increasing $\hbox{Sn}$ characteristics such as the $\hbox{Sn}$ 3d doublet and the $\hbox{Sn}$ 4d signal at $E_{\text {bin}\,\text{Sn}\,\text{3d}}\approx {490}\,\hbox{eV}$ and $E_{\text {bin}\,\text{Sn}\,\text{4d}}\approx {24}\,\hbox{eV}$, respectively. High-resolution XPS spectra of the $\hbox{Au}\,\hbox{4f}$ and $\hbox{Sn}$ 4d core levels were analyzed to resolve the system’s internal and interfacial chemical structure.

**Fig. 4:**
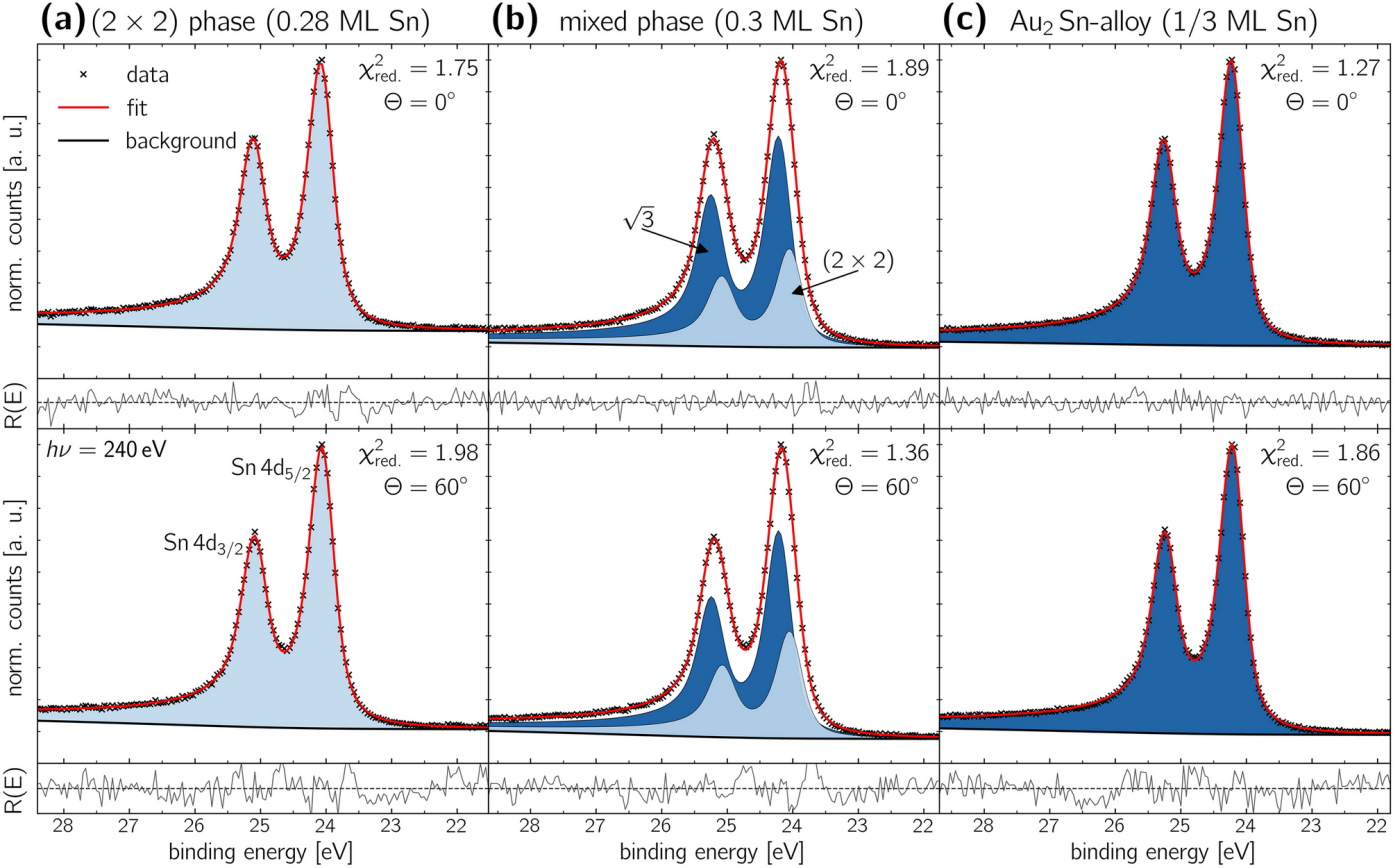
Analysis of the internal structure of the different structural phases using high-resolution XPS spectra of the $\hbox{Sn}\,\hbox{4d}$ core level. The spectra were taken at a photon energy of $\hbox{h}\nu ={240}\,\hbox {eV}$ under emission angles of $\Theta ={0}^{\circ }$ and $\Theta ={60}^{\circ }$ in the top and bottom row, respectively. Table 2 presents the corresponding fit parameters.

The chemical evolution of the $\hbox{Sn}\,\hbox{4d}$ signal is shown in Figure 4. The figure compares the spectra recorded at a polar angle of $\Theta ={0}^{\circ }$ with a higher contribution of bulk atoms in the top row, with the spectra taken at $\Theta ={60}^{\circ }$ with a greater contribution of surface atoms and higher surface sensitivity in the bottom row. The corresponding fit parameters are provided in Table 2.

Figure 4(a) displays the spectra recorded for the $(2\times 2)$-reconstruction. Data were fitted using a single Doniach-Sunjic doublet component with a spin-orbit splitting of $E_{\text {SOC}}={1.03}\,\hbox {eV}$^44^, which is in excellent agreement with the literature^45,46^. Since only one component was found, it can be assigned to the $(2\times 2)$-reconstruction. Its binding energy was determined to be $E_{\text {bin}}={24.03}\,\hbox {eV}$, which is approximately the binding energy of pure $\hbox{Sn}$ metal^47,48^. The asymmetry was found to be $\beta =0.12$, which indicates the metallic character of the valence band of this phase^24,25^. Both the relatively constant residual $\hbox{R(E)}$ as well as the obtained small $\chi ^2_{\text {red.}}$ value indicate an excellent fit quality.

With a slight increase in film thickness, as shown in Figure 4(b), the full width at half maximum (FWHM) of the $\hbox{Sn}\,\hbox{4d}$ signal increases from $\text {FWHM}_{(2\times 2)}={0.47}\,\hbox {eV}$ to $\text {FWHM}_{\text {mixed-phase}}={0.56}\,\hbox {eV}$, along with a shift of the signal’s maximum by ${100\,\hbox{meV}}$ towards higher binding energies. This shift suggests a change in the chemical composition of the layer, while the increased $\hbox{FWHM}$ indicates the presence of two distinct components.

The best fit was achieved for a model with two spectral components. In the model, approximately 1/3 of the signal’s area is attributed to the previously identified $(2\times 2)$-reconstruction (light blue). The second component is shifted by ${0.16}\,\hbox {eV}$ towards higher binding energies. It is assigned to the $(\sqrt{3}\times \sqrt{3})\text {R}{30}^{\circ }$-structure observed for this phase, and its reflexes are marked in dark blue in Figure 2(b). Both components exhibit a high asymmetry parameter of $\beta =0.12$, indicating the metallic character of their valence bands for both structural arrangements. Since we can not identify any differences in the XPS spectra between the $(2\times 2)$- and the $(2\times 2)\text {R}{8}^{\circ }$-component within our detection limit, we will no longer distinguish between these two potentially slightly different phases. From this point forward, we will refer to this phase solely as the $(2\times 2)$-reconstruction.

Figure 4(b) (bottom row) shows the spectrum measured at $\Theta ={60}^{\circ }$ emission angle. Compared to the spectrum taken at $\Theta ={0}^{\circ }$, we find no modification of the two spectral components, indicating none of the components is buried beneath the other. Most likely, both the $(2\times 2)$- and the $(\sqrt{3}\times \sqrt{3})\text {R}{30}^{\circ }$-reconstructions are located in the topmost layer, which is confirmed by our STM measurements.

With a further increase in film thickness, the best agreement is again achieved by fitting the spectrum with a single component, as shown in Figure 4(c). The fit reveals an asymmetry parameter of $\beta =0.12$, a binding energy of $E_{\text {bin}}={24.20}\,\hbox {eV}$, and an identical FWHM to the $(\sqrt{3}\times \sqrt{3})\text {R}{30}^{\circ }$-component in the mixed phase discussed above. This result strongly indicates that this component originates from the $(\sqrt{3}\times \sqrt{3})\text {R}{30}^{\circ }$ reconstruction of the ${\hbox {Au}_{2}\hbox {Sn}}$-alloy, which was already identified in the mixed phase.

**Table 2:** XPS analysis fit parameters to the chemical evolution of the $\hbox{Sn}\,\hbox{4d}$ signal shown in Figure 4. In order to simplify the reading and the table layout, the $(\sqrt{3}\times \sqrt{3})\text {R}{30}^{\circ }$-table entry was simplified to $\sqrt{3}$.

Structural phase	$\Theta$ ($^{\circ}$)	Component	$E_{\text {bin}}$ ($\hbox {eV}$)	$E_{\text {SOC}}$ ($\hbox {eV}$)	FWHM ($\hbox {eV}$)	asymmetry $\beta$	rel. area (${\%}$)
$(2\times 2)$ phase figure 4(a)	0	$(2\times 2)$	24.03	1.03	0.47	0.12	100.00
	60	$(2\times 2)$	24.04	1.03	0.47	0.12	100.00
mixed phase figure 4(b)	0	$\sqrt{3}$	24.19	1.03	0.48	0.12	68.03
		$(2\times 2)$	24.03	1.03	0.48	0.12	31.97
	60	$\sqrt{3}$	24.19	1.04	0.48	0.12	65.78
		$(2\times 2)$	24.03	1.04	0.48	0.12	34.22
$\hbox {Au}_{2}\hbox {Sn}$-alloy figure 4(c)	0	$\sqrt{3}$	24.20	1.03	0.47	0.12	100.00
	60	$\sqrt{3}$	24.19	1.03	0.47	0.12	100.00

**Fig. 5:**
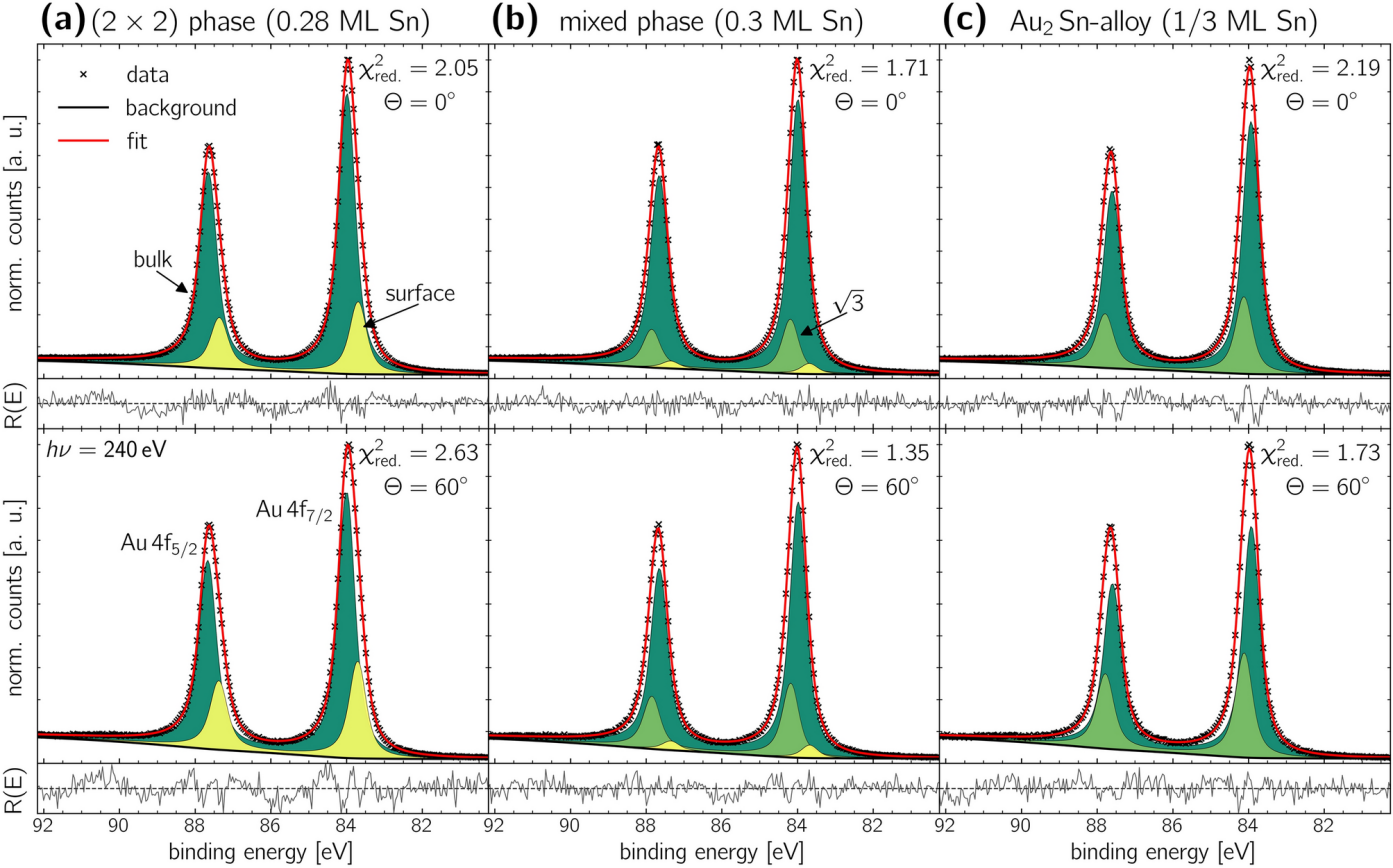
Analysis of the interface structure of the different structural phases using high-resolution XPS spectra of the $\hbox{Au}\,\hbox{4f}$ core-level signal of the substrate crystal. The spectra were taken at a photon energy of $\hbox{h}\nu ={240}\,\hbox {eV}$ under emission angles of $\Theta ={0}^{\circ }$ (top row) and $\Theta ={60}^{\circ }$ (bottom row). The corresponding fit parameters can be found in Table 3.

To investigate the chemical composition of the $\hbox{Sn}$-$\hbox{Au}$ interface, high-resolution spectra of the $\hbox{Au}\,\hbox{4f}$ core level were acquired, as shown in Figure 5. All spectra were recorded at emission angles of $\Theta ={0}^{\circ }$ and $\Theta ={60}^{\circ }$ using an excitation energy of $\hbox{h}\nu ={240}\,\hbox {eV}$. The corresponding fit parameters are listed in Table 3.

In the first step, we start with the analysis of the $(2\times 2)$-reconstruction, with its spectra shown in Figure 5(a). The best fit was achieved using a model consisting of two components that match the clean gold substrate’s FWHM, asymmetry parameter $\beta$, and binding energy $E_{\text {bin}}$. Here, we find a modified area contribution to the spectra with a reduced contribution from the surface component compared to that of the clean substrate. We find no evidence of a chemical bonding in the XPS spectra, and the surface component remains unmodified. Therefore, we conclude that the $(2\times 2)$-reconstruction exhibits no strong interaction with the substrate. Further, this conclusion is supported by the $\hbox{Sn}\,\hbox{4d}$ binding energy matching to the binding energy of metallic $\hbox{Sn}$.

To our best knowledge, the $(2\times 2)$-phase has not previously been reported. Prior studies by Maniraj et al.^20^ and Sadhukhan et al^19,43^. described periodic $\hbox{Sn}$ arrangements on $\hbox{Au(111)}$ starting at a coverage of ${1}/{3}\,\textrm{ML}$, forming a $(\sqrt{3}\times \sqrt{3})\text {R}{30}^{\circ }$-structure.

At a higher coverage of around ${2}/{3}\,\textrm{ML}$ at room temperature, an incommensurate reconstruction has been reported, referred to as the “X-Phase” by Maniraj et al.^20^, as a “$(2\times \sqrt{3})$-superstructure” by Pang et al.^17^, and as a “p$(3\times 3)\text {R}{15}^{\circ }$-reconstruction” by Sadhukhan et al.^19^. The XPS analysis by both Pang et al. and Sadhukhan et al. includes a component with a binding energy matching to that of our $(2\times 2)$-phase at $E_{\text {bin}}={24.03}\,\hbox {eV}$.

Notably, Pang et al. identified an “**S2**-component” in their analysis corresponding to a honeycomb, graphene-like $\hbox{Sn}$ arrangement, which is similar in binding energy to our $(2\times 2)$-phase. Suppose the packing density of our observed hexagonal structure is increased by adding $\hbox{Sn}$ atoms between the neighboring $\hbox{Sn}$ atoms of the $(2\times 2)$-phase. In that case, it transforms into the honeycomb structure reported by Pang et al.. Our observed hexagonal structure is characterized in detail in the STM-Section below. Therefore, our observed $(2\times 2)$-reconstruction might represent a precursor phase to a more densely packed, honeycomb-like arrangement of stanene.

Examining the high-resolution XPS spectra of the mixed phase as shown in Figure 5(b), a new component appears at a binding energy of $E_{\text {bin}}={84.21}\,\hbox {eV}$. The component, depicted in light green, is attributed to the $(\sqrt{3}\times \sqrt{3})\text {R}{30}^{\circ }$-reconstruction. Similar to the corresponding component observed in the $\hbox{Sn 4d}$ spectra (Figure 4(b)), that component is shifted to higher binding energies relative to the bulk $\hbox{Au}$ signal. Such shifts towards higher binding energies are characteristic of ${\hbox{Au}_x\hbox{Sn}}$-alloy formation^49^and have also been noted in $\hbox{AgSn}$ alloying^50^.

Typically, based on electronegativity differences, an electron transfer from $\hbox{Sn}$ to Au is expected because $\hbox{Au}$ has a higher Pauling electronegativity of 2.54 compared to 1.96 for $\hbox{Sn}$. This transfer should cause a shift of the respective component in the $\hbox{Au}\,\hbox{4f}$ signal to lower binding energies. However, in both the $\hbox{Au}\,\hbox{4f}$ and $\hbox{Sn}\,\hbox{4d}$ spectra, the $(\sqrt{3}\times \sqrt{3})\text {R}{30}^{\circ }$components shift towards higher binding energies. Egelhoff demonstrated that binding energy shifts can occur in either direction depending on the changes in valence configurations during alloying^51^.

Figure 5(c) presents the XPS spectra of the $\hbox{Au}\,\hbox{4f}$ signal to the ${\hbox {Au}_{2}\hbox {Sn}}$-alloy phase. The best fit includes two components: the bulk $\hbox{Au}$-signal and the $(\sqrt{3}\times \sqrt{3})\text {R}{30}^{\circ }$-component in dark and light green, respectively. They were already identified and discussed above in the mixed phase. Comparing measurements at different emission angles, the $(\sqrt{3}\times \sqrt{3})\text {R}{30}^{\circ }$-component increases significantly at $\Theta ={60}^{\circ }$, indicating its presence at the surface. Specifically, the area contribution to the spectrum of this component increases by $\approx {33}^{\circ }$ when varying the polar angle from $\Theta ={0}^{\circ }$ to $\Theta ={60}^{\circ }$. This increase is slightly larger than the ${30}^{\circ }$ increase observed for the surface component of both the clean $\hbox{Au}$ crystal (Figure 1(d)) and the $(2\times 2)$-reconstruction, when changing from $\Theta ={0}^{\circ }$ to $\Theta ={60}^{\circ }$ emission angle. Table 1 and Table 3 present the area contributions of the components to the respective reconstructions. This observation suggests a similar stacking order for both components, identifying the ${\hbox {Au}_{2}\hbox {Sn}}$ as an alloy in the topmost layer. Moreover, the binding energies reported here for the $(\sqrt{3}\times \sqrt{3})\text {R}{30}^{\circ }$-reconstruction is in excellent agreement with those reported by Sadhukhan et al^19,43^..

**Table 3:** Fit parameters of the XPS analysis corresponding to the $\hbox{Au}\,\hbox{4f}$ core-level signal in Figure 5. In order to simplify the reading and the table layout, the $(\sqrt{3}\times \sqrt{3})\text {R}{30}^{\circ }$-table entry was simplified to $\sqrt{3}$.

Structural phase	$\Theta$ ($^{\circ }$)	Component	$E_{\text {bin}}$ ($\hbox {eV}$)	$E_{\text {SOC}}$ ($\hbox {eV}$)	FWHM ($\hbox {eV}$)	asymmetry $\beta$	rel. area (${\%}$)
$(2\times 2)$ phase figure 5(a)	0	bulk	84.01	3.67	0.53	0.03	79.48
		surface	83.72	3.67	0.53	0.03	20.52
	60	bulk	84.00	3.67	0.53	0.03	73.27
		surface	83.71	3.67	0.53	0.03	26.73
mixed phase figure 5(b)	0	bulk	84.01	3.67	0.53	0.03	80.93
		surface	83.71	3.67	0.53	0.03	3.07
		$\sqrt{3}$	84.21	3.67	0.53	0.03	16.00
	60	bulk	84.01	3.67	0.52	0.03	74.64
		surface	83.71	3.67	0.52	0.03	3.74
		$\sqrt{3}$	84.21	3.67	0.52	0.03	21.63
$\hbox {Au}_{2}\hbox {Sn}$-alloy figure 5(c)	0	bulk	84.00	3.67	0.52	0.03	76.63
		$\sqrt{3}$	84.19	3.67	0.53	0.03	23.37
	60	bulk	84.01	3.67	0.52	0.03	68.88
		$\sqrt{3}$	84.20	3.67	0.53	0.03	31.12

### STM

**Fig. 6:**
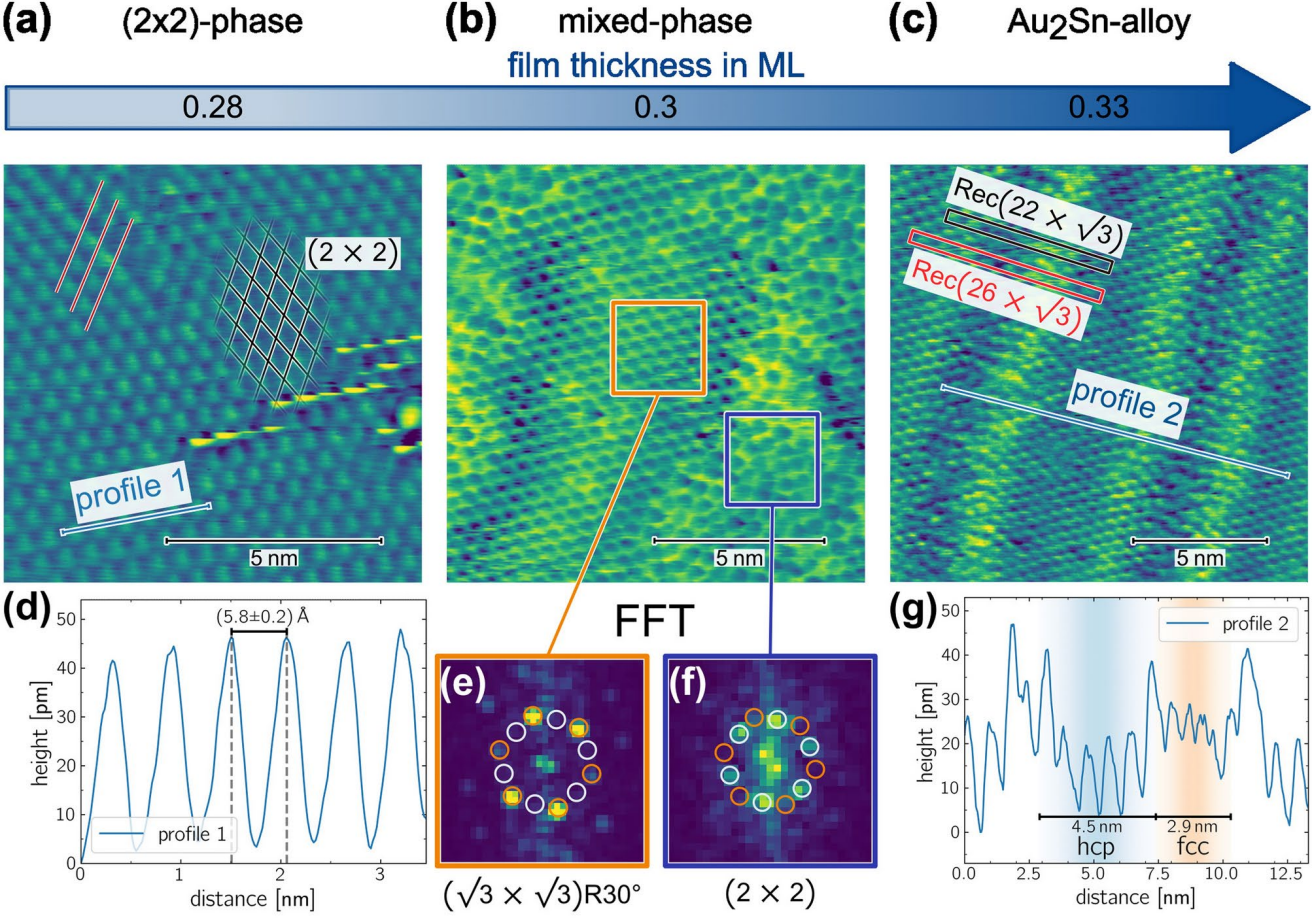
Structure analysis of the different phases using STM at room temperature. **(a)** Small-area STM image of the $(2\times 2)$-phase recorded with $U_{\text {bias}} = {0.75}\,\hbox{V}$ and $I_{\text {tunnel}} = {176}\,\hbox{pA}$. The corresponding line profile is shown in (d). **(b)** The mixed phase was recorded with $I_{\text {tunnel}} = {22.3}\,\hbox{pA}$. It consists of alternating stripes of a well-reconstructed $(\sqrt{3} \times \sqrt{3})\text {R}30^{\circ}$- and distorted stripes of the $(2\times 2)$-phases. FFT’s of the areas to the orange and blue marked boxes for the $(\sqrt{3} \times \sqrt{3})\text {R}30^{\circ}$- and $(2\times 2)$-phases are shown in (e) and (f), respectively. **(c)** Alternating stripes of the local $(\sqrt{3}\times \sqrt{3})\text {R}{30}^{\circ}$-reconstruction reveal similarities to the herringbone reconstruction of the clean ${\hbox{Au}(111)}$ surface. A corresponding $\text {Rec}(22\times \sqrt{3})$ herringbone unit cell is marked in black, while a $\text {Rec}(26\times \sqrt{3})$ unit cell is highlighted in red. A line profile is shown in (g). **(d)** Line profile of the $(2\times 2)$-phase. **(e)** and **(f)** display the FFT of the marked boxes in (b). A well-ordered $(\sqrt{3}\times \sqrt{3})\text {R}{30}^{\circ}$-structure is shown in (e), with its spot pattern indicated by orange circles, marked by white circles are the expected positions of the $(2\times 2)$-phase. In (f), a hexagonal Fourier pattern is observed as well, which is rotated by ${30}^{\circ}$ to the $(\sqrt{3}\times \sqrt{3})\text {R}{30}^{\circ}$-structure, indicating that it originates from an unrotated $(2\times 2)$-reconstruction relative to the $\hbox{Au}$ surface. **(g)** shows the line profile corresponding to the striped $(\sqrt{3}\times \sqrt{3})\text {R}{30}^{\circ}$-reconstruction in (c). The indicated width of the fcc and hcp stacking are obtained from our XPD analysis below.

We performed STM measurements to investigate the structural evolution of $\hbox{Sn}$ on ${\hbox{Au}(111)}$ in real space. The STM images are shown in Figure 6.

At a coverage of approximately $0.28\,\textrm{ML}$ a hexagonal arrangement of $\hbox{Sn}$ with a $(2\times 2)$-periodicity relative to the ${\hbox{Au}(111)}$ substrate is observed as shown in Figure 6(a). Often, this $(2\times 2)$-reconstruction coexists with regions of unordered $\hbox{Sn}$ deposited at the $(2\times 2)$ surface, as presented in Fig. S1 of the Supplementary information. Dislocation lines are frequently present, showing two domains of the ${\hbox{Au}(111)}$-reconstruction that are shifted by half of the lattice constant of the $(2 \times 2)$-phase. The shift between neighboring domains is indicated by red lines in the top left of Figure 6(a). Dislocation lines with widths of $\approx a_{\text {Au}(111)}$ and $\approx 5\cdot a_{\text {Au}(111)}$ are observed, with the latter containing two dense-packed $\hbox{Sn}$-atom rows, as depicted in Supplementary Fig. S2.

Averaging six different height profiles, e.g. **profile 1** in Figure 6(a), which is plotted in Figure 6(d), the distance between adjacent $\hbox{Sn}$ atoms was measured. This yields a $\hbox{Sn}$-atom distance of $a_{\text {Sn}, (2\times 2)} = {{5.8}{\pm }{0.2}}{\text{\AA }}$ which is closely matching the expected lattice constant of the $(2\times 2)$-reconstruction on the unreconstructed ${\hbox{Au}(111)}$ surface of $2 \cdot a_{\text {Au}(111)} = {5.764}{\text{\AA }}$.

The mixed phase in Figure 6(b) exhibits a stripe-like pattern, characterized by alternating stripes of a well-ordered $(\sqrt{3} \times \sqrt{3})\text {R}30^{\circ }$-phase and often distorted stripes of the $(2\times 2)$-phase. The stripe widths range from $\approx {5}\,\hbox {nm}\, \hbox{-}10 \,\hbox {nm}$. Fast-Fourier Transformation (FFTs) of the blue and orange marked areas in Figure 6(b), calculated with Gwyddion^52^ are shown in Figures 6(e) and (f). The FFT of the $(\sqrt{3} \times \sqrt{3})\text {R}30^{\circ}$-region (orange) shows sharp, hexagonally arranged spots, marked by a light green hexagon indicating the $(\sqrt{3} \times \sqrt{3})\text {R}30^{\circ}$-orientation. In comparison, the FFT of the $(2\times 2)$-region shows spots that no longer align with the corners of the hexagon, as depicted in Figure 6(f). In contrast, these spots are located along the middle of the hexagon’s sides, indicating a clear ${30}^{\circ}$-rotation between the two structural arrangements.

In the STM image of Figure 6(c), a striped pattern of the ${\hbox {Au}_{2}\hbox {Sn}}$-alloy phase was observed, being similar to the herringbone reconstruction of clean ${\hbox{Au}(111)}$. As revealed by our XPD analysis in Section XPD, the striped structure consists of alternating hcp- and fcc-stacked stripes, separated by soliton walls, which appear as brighter stripes in the STM image. In Figure 6(g), the line profile shows the different stacking order widths obtained from our XPD analysis, which agrees well with our STM measurements. Significantly, the fcc region often shows a relatively high degree of remaining disorder. Sometimes, small areas or single hexagons matching in their lattice constant to the $(2\times 2)$-reconstruction are observed. Reminiscents of the $(2\times 2)$-reconstruction in the ${\hbox {Au}_{2}\hbox {Sn}}$-alloy phase could explain the observed distortions in the STM image as reported by Shah et al^16^..

Additionally, the $\text {Rec}(22\times \sqrt{3})$ unit cell of the herringbone reconstruction and a $\text {Rec}(26\times \sqrt{3})$unit cell as suggested by Shah et al^16^. are plotted in black and red in Figure 6(c), respectively. The periodicity of the alternating stripes matches more closely to the $\text {Rec}(26\times \sqrt{3})$-structure.

To resolve whether the ${\hbox {Au}_{2}\hbox {Sn}}$-alloy adopts the herringbone reconstruction as suggested by Maniraj et al^15^. or whether a similar but larger $\text {Rec}(26\times \sqrt{3})$ unit cell as proposed by Shah et al^16^. is realized, we performed an additional XPD analysis of the striped ${\hbox {Au}_{2}\hbox {Sn}}$-alloy.

### XPD

To determine the structure of the ${\hbox {Au}_{2}\hbox {Sn}}$ surface alloy, we conducted XPD measurements and simulations of the $\hbox{Au}\,\hbox{4f}$ and $\hbox{Sn}\,\hbox{4d}$ core-level signals. The data analysis of the experimental data started with careful background removal. A threefold rotational symmetry operation and a mirror symmetry at $\Phi ={30}^{\circ}$ were applied to all XPD patterns.

**Fig. 7:**
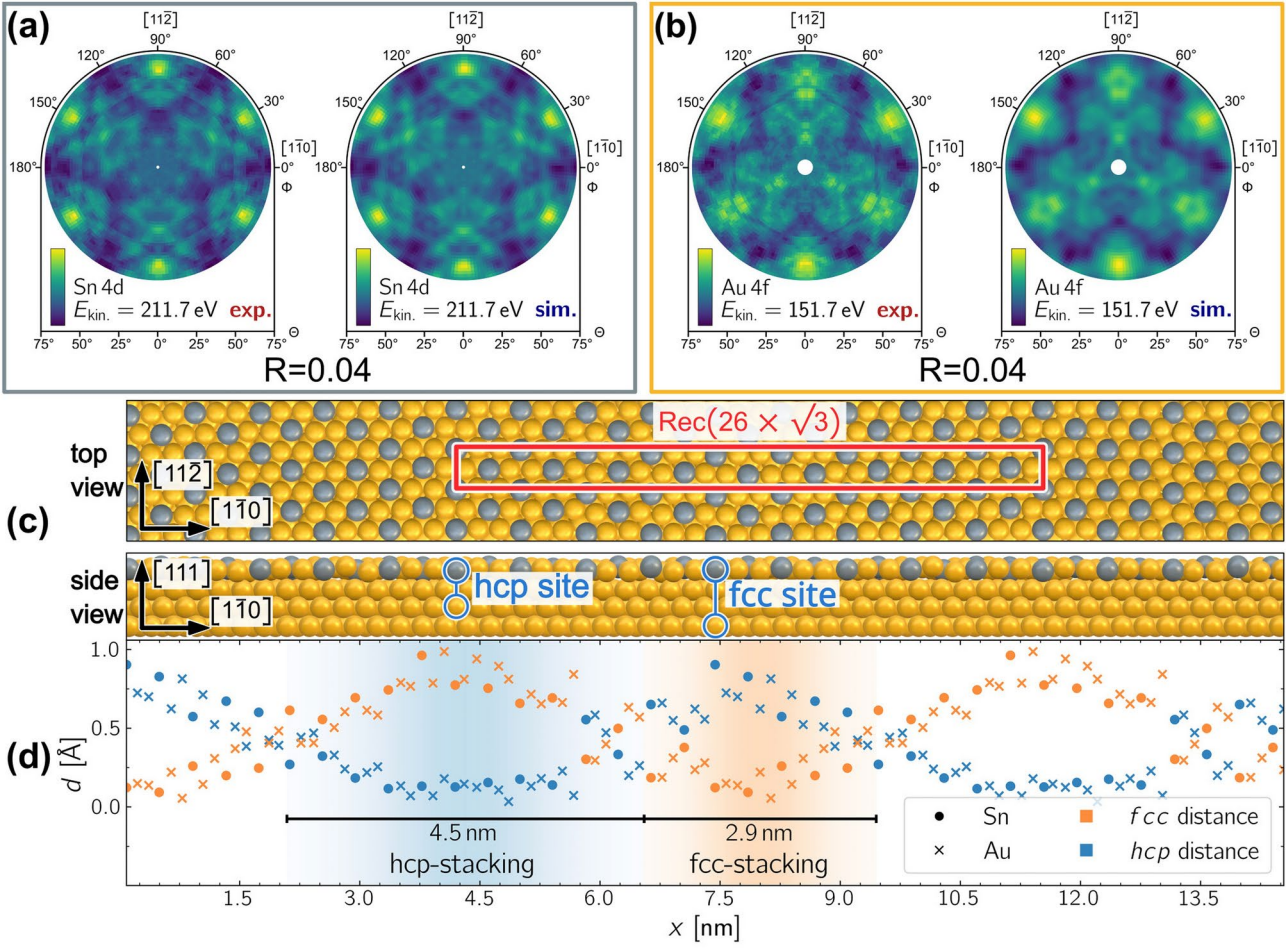
XPD analysis of the $\text {Rec}(26\times \sqrt{3})$-phase. **(a)** Experimental (left) and simulated (right) XPD patterns for $\hbox{Sn}\,\hbox{4d}$ photoelectrons with a kinetic energy of $E_{\text {kin.}} = {211.7}\,\hbox {eV}$. **(b)** Same as in (a) for $\hbox{Au}\,\hbox{4f}$ signal with $E_{\text {kin.}} = {151.6}\,\hbox {eV}$. Simulations for both patterns independently resulted in a $\text {Rec}(26\times \sqrt{3})$-reconstruction. The agreement between simulation and experiment for both patterns yielded an R-factor of $R = 0.04$ each. The obtained structure is displayed in **(c)**. **(d)** shows the lateral distance from the ideal fcc and hcp stacking sites for the atoms in the top layer of the unit cell. Similar to the herringbone reconstruction of the clean $\hbox{{Au}(111)}$ surface, alternating hcp and fcc stacking stripes are observed. The width of the hcp stripes, measuring $\approx {4.5}\,\hbox {nm}$, is larger than the fcc stripes with $\approx {2.9}\,\hbox {nm}$.

Figure 7 illustrates the XPD analysis of the ${\hbox {Au}_{2}\hbox {Sn}}$ striped alloy phase. An initial structural model was developed based on a $(\sqrt{3}\times \sqrt{3})\text {R}{30}^{\circ}$-reconstruction, where every third atom in the topmost layer of the $\hbox{{Au}(111)}$ crystal was replaced by an $\hbox{Sn}$ atom, consistent with our LEED and STM results. Additionally, strain variations of $\pm {10}{\%}$ along the $[1\overline{1}0]$-direction and minor adjustments to the positions of individual atoms in all directions within the two topmost layers were allowed. Using a genetic algorithm, we optimized this initial structural model. Two *independent* XPD structure research calculations for the $\hbox{Au}\,\hbox{4f}$ and $\hbox{Sn}\,\hbox{4d}$ core-level signals were performed. The outcomes of these independently obtained results are compared in Figures 7(a) and (b). Both simulated patterns match excellently with the experimental data, as indicated by the low R-factor of $R=0.04$. Notably, both simulations converged on the rather large unit cell of $\text {Rec}(26\times \sqrt{3})$. Each simulation contained the whole set of atoms in the surface structure; the diffraction patterns in Figures 7(a) and (b) reflect the diffraction of photoelectrons emitted from the $\hbox{Sn}\,\hbox{4d}$- and $\hbox{Au}\,\hbox{4f}$-orbital, respectively. The differences between the two independently derived surface structures were minimal, with variations of $\le {5}\,\hbox{pm}$ in the $[1\overline{1}0]$ and $[11\overline{2}]$ directions and $\le {30}\,\hbox{pm}$ in the $[111]$-direction, respectively.

In Figure 7(d), each atom’s in-plane distance $d$ to the nearest ideal fcc and hcp stacking site is plotted in orange and blue, respectively. The atoms’ positions oscillate between fcc and hcp stacking along the $[1\overline{1}0]$-direction. Atoms are assigned to the hcp region if closer to an hcp site than an fcc site, and vice versa. In transition regions, where the assignment to a stacking site is ambiguous, the atoms are assigned towards the neighboring regions, following a similar approach as used for soliton walls in the herringbone reconstruction of clean $\hbox{{Au}(111)}$^42,53,54^, where no width is assigned to the soliton wall itself. The observed disorder in the transition regions may also arise from allowed local variations in the XPD simulation. Notably, a similar striped-like phase was found for the ${\hbox{Ag}_{2}\hbox {Ge}}$ alloy formed on top of $\hbox{Ag(111)}$ after the deposition of $\approx {1}/{3}\,$ $\hbox{ML Ge}$, resulting in alternating hcp and fcc stripes with a $c(31 \times \sqrt{3})$-supercell^55^.

The hcp- and fcc-regions of the herringbone reconstruction of clean $\hbox{{Au}(111)}$ have widths of ${2.8}\,\hbox {nm}$ and ${3.8}\,\hbox {nm}$, respectively^42^. In contrast, for the ${\hbox {Au}_{2}\hbox {Sn}}$-alloy, the hcp-region is wider than the fcc-region, with measured widths of $\approx {4.5}\,\hbox {nm}$ and $\approx {2.9}\,\hbox {nm}$, respectively. As highlighted in Figure 7(g), these findings align well with our STM results.

**Fig. 8:**
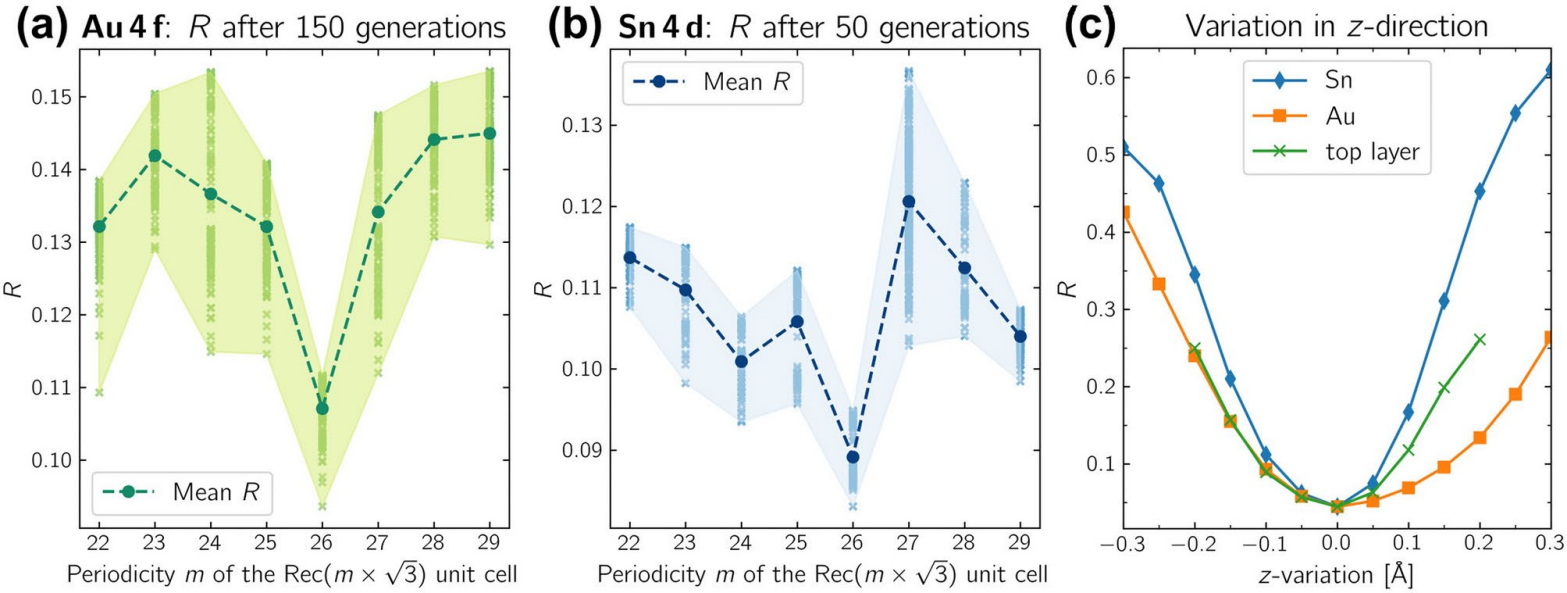
The R-factor minimum was calculated for different periodicities *m* of the $\text{Rec}( m \times \sqrt{3} )$ rectangular unit cell. The R-factors of the $\hbox{Au}\,\hbox{4f}$ signal **(a)** and the $\hbox{Sn}\,\hbox{4d}$ signal **(b)** were independently calculated, yielding the same minimum at a $m=26$ periodicity in the $[1\overline{1}0]$-direction. **(c)** confirms the R-factor minimum ($R=0.04$) against variations in *z*-direction.

Furthermore, we checked the stability of the R-factor minimum. The genetic algorithm was set to a fixed periodicity $m$ in the $[1\overline{1}0]$-direction relative to the unreconstructed $\hbox{{Au}(111)}$ surface, without any other structure variations to the initial test structure. The genetic algorithm was run *independently* for the $\hbox{Au}\,\hbox{4f}$ and $\hbox{Sn}\,\hbox{4d}$ XPD patterns. The genetic algorithm was initialized and run three times for each fixed periodicity *m*, where each generation consists of 60 individuals, to enhance statistical reliability.

Figure 8(a) and (b) display the R-factors to the $\hbox{Au}\,\hbox{4f}$- and $\hbox{Sn}\,\hbox{4d}$-pattern obtained after 150 and 50 generations, respectively. In the Figure, the dots represent the average R-factor in dependence of the periodicity *m*, with the light-color shaded area indicating the range of R-factors obtained.

For the $\hbox{Au}\,\hbox{4f}$ and ${\hbox{Sn}\,\hbox{4d}}$ patterns, the lowest R-factor and best average R-factor were independently achieved at a periodicity of $m=26$. This confirms the stability of the long-range $\text {Rec}(26\times \sqrt{3})$-periodicity in the $[1\overline{1}0]$ direction derived from our XPD simulations.

In Figure 8(c), the *z*-location of the $\hbox{Sn}$- and ${\hbox {Au}}$-atoms relative to the final structure model is plotted in blue and orange, respectively. The result of the *z*-variation of the complete ${\hbox {Au}_{2}\hbox {Sn}}$-alloy layer is plotted in green. The respective R-factors demonstrate a well-defined minimum, confirming the result and validating the robustness of the obtained structure. Additionally, we tested various preparation parameters by varying the substrate temperature and deposition rate. A list of all tested preparation conditions, including a comparison with the parameters reported by Maniraj et al. and Shah et al., is provided in the Supplementary in Tab. S3. Regardless of the preparation parameters used, the same diffraction pattern was consistently observed in LEED. A detailed analysis of the LEED pattern, following a similar approach to that conducted by Shah et al., is presented in Supplementary Fig. S4. Our structural analysis therefore confirms the $\text {Rec}(26\times \sqrt{3})$unit cell as proposed by Shah et al^16^..

## Conclusion

We have investigated the structural evolution of epitaxially grown submonolayer $\hbox{Sn}$ on $\hbox{{Au}(111)}$. Using LEED and high-resolution core-level photoelectron spectroscopy of the $\hbox{Au}\,\hbox{4f}$- and $\hbox{Sn}\,\hbox{4d}$-orbitals, we could distinguish structurally and chemically between three distinct phases. Initially, a hexagonal $(2\times 2)$-phase forms at a coverage of approximately $0.28\,\textrm{ML}$ which is not chemically bound to the substrate as XPS indicates. A slight increase in coverage to around $0.3\,\textrm{ML}$ leads to a mixed phase, consisting of both the $(2\times 2)$- and $(\sqrt{3} \times \sqrt{3})\text {R}{30}^{\circ }$-reconstructions. STM results confirm an alternating striped-like coexistence of these two phases. At a film thickness of about $0.33\,\textrm{ML}$, a sharp $(\sqrt{3} \times \sqrt{3})\text {R}{30}^{\circ }$-reconstruction is observed in LEED, corresponding to a ${\hbox {Au}_{2}\hbox {Sn}}$ surface alloy, which also exhibits a striped-like pattern in STM.

Our XPD measurements and simulations reveal an unusually large $\text {Rec}(26\times \sqrt{3})$ unit cell, similar to the herringbone reconstruction of bare $\hbox{{Au}(111)}$. In this ${\hbox {Au}_{2}\hbox {Sn}}$-structure, 27 atoms of the surface alloy layer align with 26 substrate atoms along the $[1\overline{1}0]$ direction, resulting in a lateral compression of approximately ${3.7}{\%}$ in the topmost layer and to an alternating fcc and hcp stacking of the alloy layer.

Our analysis clarifies the previously debated size of the unit cell in the ${\hbox {Au}_{2}\hbox {Sn}}$-alloy layer. It reveals the previously unknown $(2\times 2)$-reconstruction, which could provide insights into $\hbox{Sn}$-reconstructions at higher coverages on $\hbox{{Au}(111)}$. Furthermore, our detailed structural analysis of the ${\hbox {Au}_{2}\hbox {Sn}}$-alloy may contribute to the preparation of freestanding stanene on $\hbox{{Au}(111)}$ and enhances the fundamental understanding of the growth of strain-free single-atomic honeycomb 2D materials on noble metal surface alloys.

## Methods

Sample preparation and all measurements were performed *in-situ* in two separate ultra-high vacuum (UHV) chambers, each maintaining a base pressure of $p \le {1} \times {10}^{-10}\,\hbox {mbar}$. Both chambers were equipped with a sputter gun, a heating stage, and a 4-grid LEED system. To ensure nearly identical prepared structural phases in both chambers, the preparation procedure was kept consistent and was only slightly adapted to accommodate the chamber geometries. Each resulting reconstruction was carefully verified for its periodicity using LEED. Auger Electron Spectroscopy (AES) measurements were also conducted with the 4-grid LEED setup in both chambers, and the resulting signal intensities were compared to confirm consistency. As observed by LEED, the high sensitivity of the different structural phases to film thickness allowed a precise calibration of the preparation process to accommodate different chamber geometries.

The well-reconstructed $\hbox{Au(111)}$ surface was prepared by repeated cycles of Ar-ion bombardment at $E_{\text {kin}} = {1000}\,\hbox {eV}$, followed by annealing at $T=950\,\hbox{K}$. A detailed description of the preparation cycles used to prepare the clean $\hbox{{Au}(111)}$ are given in Tab. S1 in the Supplementary. Epitaxial growth of the various structural tin phases was achieved using physical vapor deposition (PVD). The evaporation rate was estimated at approximately ${3}\,{{\text{\AA }}{h}^{-1}}$, as determined by quartz crystal microbalance measurements. The thickness of the overlayer film was further confirmed using XPS survey spectra, following the method described by Zemlyanov et al^56^.. In this paper, we define film thicknesses in terms of monolayers (ML), where $1\,\textrm{ML}$ of $\hbox{Sn}$ corresponds to the atomic density of a $\hbox{{Au}(111)}$ plane, which is ${1.391 \times {10}^{15}}\, {\text {atoms}}/{{\hbox {cm}^{2}}}$. Therefore, ${1}/{3}\,\textrm{ML}$ of $\hbox{Sn}$ is grown in $\approx {20}\,\hbox{min}$.

The XPS and XPD measurements were conducted at the endstation of beamline 11 at the DELTA electron storage ring, TU Dortmund University. This beamline provides linearly polarized soft x-ray radiation from the U55 undulator, spanning an energy range of ${50}\,\hbox {eV} \le \hbox{h}\nu \le {1500}\,\hbox {eV}$, and is freely tunable using a plane-grating monochromator^57^. The endstation is equipped with a 5-axis manipulator, allowing for movement of the sample along the *x*-, *y*-, and *z*-axes, as well as continuous rotations of azimuthal ($\Phi$) and polar axis ($\Theta$) relative to the surface normal. XPS and XPD data were acquired using a hemispherical CLAM IV analyzer.

Survey spectra were collected with a pass energy of ${50}\,\hbox {eV}$ and an energy increment step size $\Delta E={1.22}\,\hbox {eV}.$ High-resolution spectra and valence-band spectra were taken at pass energy of ${5}\,\hbox {eV}$ and with an increment of $\Delta E={41}\,\hbox {meV}$. Survey spectra, high-resolution spectra, and valence-band spectra were recorded at $\hbox{h}\nu ={700}\,\hbox {eV}$, $\hbox{h}\nu ={240}\,\hbox {eV}$, and $\hbox{h}\nu ={52.5}\,\hbox {eV}$, respectively. All core-level high-resolution and survey spectra were collected at both normal and high emission angles, with polar angles $\Theta = {0}^{\circ}$ and $\Theta = {60}{^{\circ}}$, respectively. At normal emission with a polar angle of $\Theta = {0}{^{\circ}}$ the photoelectron signal contains major contributions from below the surface while at high polar angles at $\Theta = {60}{^{\circ}}$ contributions from the surface are dominating. A comparison of the bulk- and surface-enhanced spectra allows the extraction of structure information of the interface and surface.

The high-resolution spectra were processed using the python-based software LG4X-V2^44^, which utilizes the LMFIT^58^ and lmfitxps^59 ^python packages for fitting via the Levenberg-Marquardt algorithm. Each spectrum was modeled using a Doniach-Sunjic distribution^60^, with $\beta$ as an asymmetry parameter and convoluted with a Gaussian distribution. The fit model function includes a Tougaard background, allowing simultaneous optimization of the background and peak model parameters.

Tables 1 to 3 present the obtained fit parameters for the $\hbox{Au}\,\hbox{4f}_{7/2}$- and $\hbox{Sn}\,\hbox{4d}_{5/2}$-doublet-signals. Identical fit parameters were applied to both peaks of a doublet. The energy shift $E_{\text {SOC}}$ due to spin-orbit coupling between two peaks of a doublet is listed in the tables. All binding energies refer to the Fermi energy level. The Fermi-energy was determined in a fit applying the LG4X-V2-software^44^. At the beginning and end of each data set, high-resolution spectra of the Fermi edge were taken. The Gaussian broadening introduced by the experimental setup-specifically due to the excitation light source, thermal effects, and the spectrometer-was determined by fitting the Fermi edge. The resulting value for the Gaussian contribution to the $\hbox{FWHM}$ was then applied in all high-resolution XPS fits, while allowing only minor adjustments of less than $5\%$ during the fitting process. As an example, a fitted Fermi edge along with the corresponding parameters is shown in Fig. S3 and Tab. S2 of the Supplementary Material. The normalized residual *R*(*E*) provides the fit quality presented for each fitted spectrum in the bottom part of the Figures. Additionally, the reduced chi-squared $\chi ^2_{\text {red.}}$ is reported as an indicator of the goodness of the fit.

The XPD measurements were performed to investigate the structural arrangement of the ${\hbox {Au}_{2}\hbox {Sn}}$-alloy phase. These measurements rely on the intensity modulation of the photoelectron signal due to interference effects^61,62^. The interference pattern is created when the spherical electron wave propagates through the crystal lattice and is diffracted by neighboring atoms, resulting in angle-dependent intensity variations. To map the interference pattern of the $\hbox{Sn}\,\hbox{4d}$ core-level electrons, high-resolution XPS spectra were recorded over a polar angle range of ${2}{^{\circ}} \le \Theta \le {72}{^{\circ}}$ in steps of $\Delta \Theta = {2}{^{\circ}}$ and an azimuthal range spanning ${0}{^{\circ}} \le \Phi < {360}{^{\circ}}$ with increments of $\Delta \Phi = {1.8}{^{\circ}}$, resulting in a total of 7200 individual XPS spectra. The XPD measurements for the $\hbox{Au}\,\hbox{4f}$ electrons were conducted using the same parameters, except for a reduced polar angle range of ${6}{^{\circ}} \le \Theta \le {72}{^{\circ}}$ due to limited beam time. XPD patterns were obtained using an excitation energy of $\hbox{h}\nu = {240}\,\hbox {eV}$. The presented XPD patterns were derived by integrating the intensities of the $\hbox{Au}\,\hbox{4f}_{7/2}$- and $\hbox{Sn}\,\hbox{4d}_{5/2}$-signals, respectively. In addition, an anisotropy function was applied to the XPD pattern for each polar angle $\Theta$, defined as^63^:

1 $$\begin{aligned} \chi (\Theta , \Phi ) = \frac{I(\Theta , \Phi ) - \overline{I(\Theta )}}{\overline{I(\Theta )}}, \end{aligned}$$

where $I(\Theta , \Phi )$ is the intensity measured at emission direction defined by angles $\Theta$ and $\Phi$, and $\overline{I(\Theta )}$ represents the average intensity over all azimuthal angles for each $\Theta$. The resulting XPD patterns were further processed, assuming a threefold symmetry. All kinetic energies applied in simulations were based on the peak center of measured XPS spectra. This procedure yielded kinetic energies of $E_{\text {kin}} \approx {151.7}\,\hbox {eV}$ and $E_{\text {kin}} \approx {211.7}\,\hbox {eV}$ for the $\hbox{Au}\,\hbox{4f}_{7/2}$- and $\hbox{Sn}\,\hbox{4d}_{5/2}$-signal, respectively. The simulations were performed using the EDAC algorithm^64^, which enables multiple scattering calculations of the photoelectron diffraction patterns. The agreement between experimental and simulated patterns was used to evaluate the consistency between the sample structure and the proposed model structure^65^. Pendry’s reliability factor (R-factor)^66^ quantifies the agreement between the experimental and simulated XPD patterns:

2 $$\begin{aligned} \textrm{R} = \frac{\sum _{\Theta , \Phi } \left[ \chi _{\exp }(\Theta , \Phi ) - \chi _{\text {sim}}(\Theta , \Phi )\right] ^2}{\sum _{\Theta , \Phi } \left[ \chi _{\exp }^2(\Theta , \Phi ) + \chi _{\text {sim}}^2(\Theta , \Phi )\right] }. \end{aligned}$$

The R-factor ranges from $0 \le \textrm{R} \le 2$, where $\textrm{R} = 0$ indicates perfect agreement, $\textrm{R} = 1$ indicates independent patterns, and $\textrm{R} = 2$ corresponds to anticorrelated patterns.

A genetic algorithm was employed to minimize the R-factor and thus to match the diffraction pattern of the test structure to the experimental result^67,68^. The initial test structure consisted of a slab containing five atom layers of gold bulk and one alloy layer, where every third gold atom was replaced by a tin atom, forming a $(\sqrt{3} \times \sqrt{3})\text {R}{30}{^{\circ}}$-alloy layer. The lattice constant of the $\hbox{{Au}(111)}$ surface was set at $a_1=a_2 = {2.882}\,{\text{\AA }}$^69^. A supercell size of ${26}\,\hbox{nm} \times {4}\,\hbox{nm}$ was used to account for long-range periodicities. During the simulations, the locations of the atoms in the two uppermost layers - the alloy layer and the first gold-bulk layer - were allowed to vary. Periodic boundary conditions were applied for both the local $(\sqrt{3} \times \sqrt{3})\text {R}{30}{^{\circ }}$-structure and the long-range $\text {Rec}(m \times \sqrt{3})\text {R}{30}{^{\circ }}$ strain. The STM measurements were performed using a SCALA RT-STM at room temperature. Chemically etched with $20 \%$ $\hbox{NaOH}$, Tungsten tips were further sharpened *in-situ* by applying voltage pulses to enhance image quality. All measurements were performed in constant current mode, with tunneling current ($I_{\text {tunnel}}$) and bias voltage ($U_{\text {bias}}$) varied between measurements. The specific STM image parameters are presented in the Figure captions. The scan area was adjusted for each measurement while keeping the resolution of $400\,\text {px}\times 400\,\text {px}$ constant. Data processing, including plane leveling, minor drift correction, and Fourier analysis, was performed using Gwyddion software^52^.

## Supplementary Information

Supplementary Information.

## Data Availability

The data supporting this study’s findings are available from the corresponding author upon reasonable request. Due to their large size and specific formatting, the data files are not hosted in a public repository but can be provided for research purposes. The underlying code for the XPD simulations is not publicly available but may be made available to qualified researchers on reasonable request from the corresponding author.
