# Supplementary material for: Structural analysis of [image] on [image] at low coverages: Towards the [image] surface alloy with alternating fcc and hcp domains
Source: Sci Rep. 2025 Mar 7;15:7953. doi: 10.1038/s41598-025-91733-2 (PMC11889230; doi:10.1038/s41598-025-91733-2)
Supplement: Supplementary file 1 — Supplementary Information. [file 41598_2025_91733_MOESM1_ESM.pdf]

Structural analysis of Sn on Au(111) at low coverages: Towards the Au_2Sn surface alloy with alternating fcc and hcp domains (Supplementary Information)

Julian A. Hochhaus[1,2,*], Stefanie Hilgers[1,2], Marie Schmitz[1,2], Lukas Kesper[1,2], Ulf Berges[1,2], and Carsten Westphal[1,2]

[1]Department of Physics, TU Dortmund University, Otto-Hahn-Str. 4a, 44227 Dortmund, Germany
[2]DELTA, Center for Synchrotron Radiation, TU Dortmund University, 44227 Dortmund, Germany
[*]julian.hochhaus@tu-dortmund.de

Table S1. Preparation parameters of the clean Au (111) substrate. The first two cycles are applied only if the crystal was freshly inserted into the UHV chamber.

Cycle	Process Step	Details	Pressure in UHV Chamber
First Two Cycles	Sputtering	Performed for 1 hour using Ar-ions with a kinetic energy of $E = 1000\,\mathrm{eV}$; ion current at sample: $I_{\mathrm{Ion}} \approx 3\,\mu\mathrm{A}$	$4 \times 10^{-6}\,\mathrm{mbar}$ (Ar-atmosphere)
	Annealing	Temperature increased linearly over 10 min to $T_{\mathrm{final}} \approx 950\,\mathrm{K}$; held at T_{final} for 120 min; temperature decreased parabolically over 20 min	$\leq 5 \times 10^{-10}\,\mathrm{mbar}$
Repeated Cycles (at least 5 times)	Sputtering	Performed for 1 hour using Ar-ions with a kinetic energy of $E = 1000\,\mathrm{eV}$; ion current at sample: $I_{\mathrm{Ion}} \approx 1.2\,\mu\mathrm{A}$	$2 \times 10^{-6}\,\mathrm{mbar}$ (Ar-atmosphere)
	Annealing	Temperature increased linearly over 10 min to $T_{\mathrm{final}} \approx 920\,\mathrm{K}$; held at T_{final} for 120 min; temperature decreased parabolically over 20 min	$\leq 3.5 \times 10^{-10}$ millibar

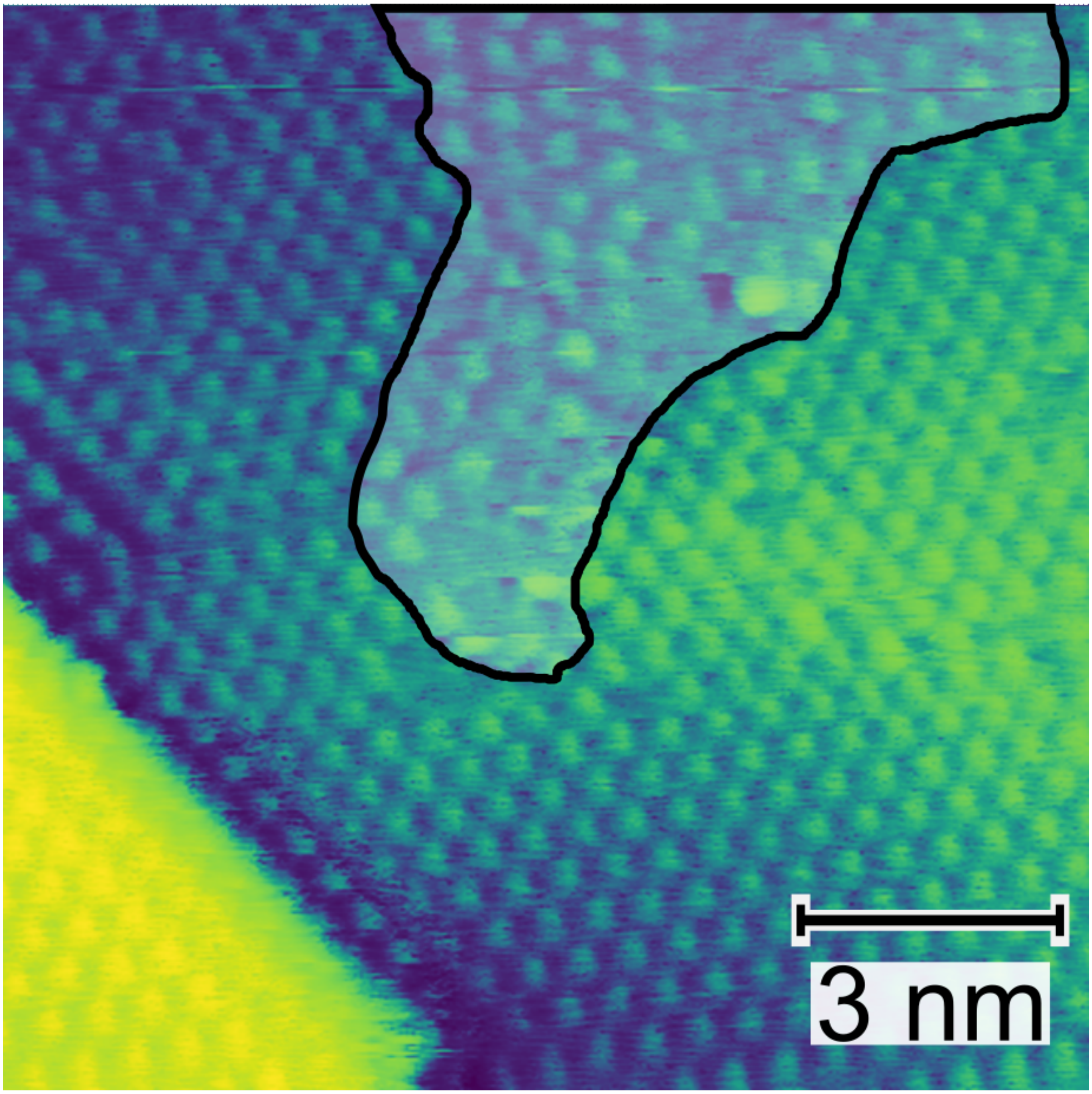

Figure S1. Coexistence of the well-ordered (2×2)-reconstruction with regions of unordered Sn, outlined by a black border.

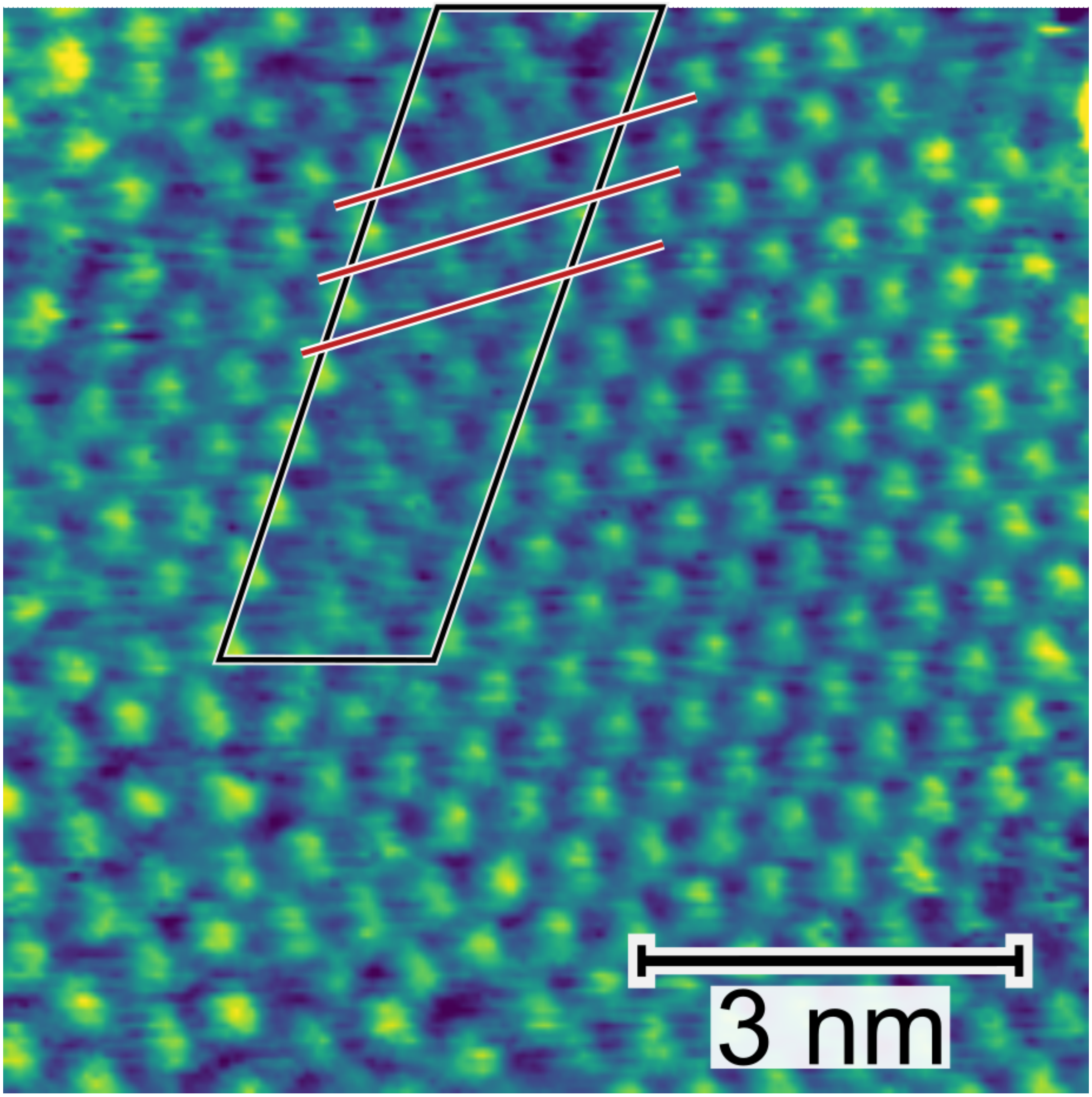

Figure S2. Separation line in the well-ordered (2×2)-reconstruction with a width of $\approx 5 \cdot a_{\mathrm{Au}(111)}$ containing two dense-packed Sn-atom rows. In contrast to the dislocation line shown in Fig. 6 (a) in the main manuscript, no resulting dislocation shift is observed as indicated by the red lines. The two dense-packed Sn-atom rows are aligned with the neighbouring reconstruction as well.

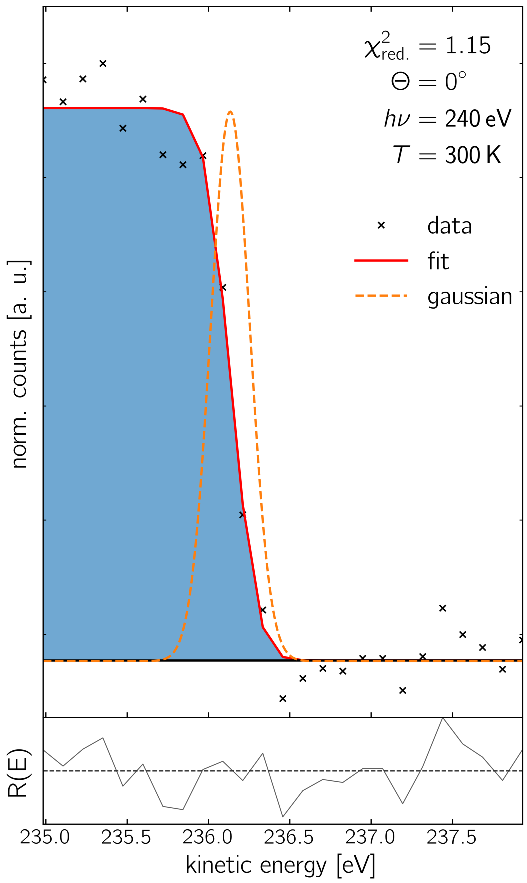

Figure S3. Fit of the Fermi edge of the (2×2)-reconstruction using the `FermiEdgeModel` from the *lmfitxps*-package[1]. The Fermi edge was measured both before and after recording each dataset to ensure accurate determination of binding energies. The Gaussian width (depicted with an orange dashed line) was used to evaluate the energy resolution of the experimental setup.

Table S2. Parameters of the Fermi edge fit using the `FermiEdgeModel` from the *lmfitxps*-package[1], as shown in Figure S3. The `FermiEdgeModel` employs a convolution of a Fermi step edge with a Gaussian to account for broadening effects originating from the excitation source and broadening introduced by the analyzer/spectrometer.

Parameter	Value (eV)	Comment/Remark
center	236.134	Center of the Fermi edge. Used for binding energy calibration
kT	0.024	$k_B T$ at room temperature, effective temperature parameter for the Fermi-Dirac distribution.
sigma	0.123	Standard deviation σ of the gaussian profile (Gaussian broadening)
FWHM	0.289	Gaussian contribution to the FWHM ($\mathrm{FWHM_{Gaussian}} = 2\sqrt{\ln(2)}\,\sigma$), used for all fits of XPS high-resolution spectra in the main paper.

Table S2. Comparison of the preparation parameters used by Maniraj et al.[4], Shah et al.[3], and the tested parameter ranges in the present paper. Independent of the used preparation parameters, the LEED pattern always indicated a $\mathrm{Rec}(26 \times \sqrt{3})$-periodicity as analysed in Figure S4.

Parameter	Maniraj et al.[4]	Shah et al.[3]	Present paper
Evaporation rate	not given, Sn in crucible at 1100 K	not given	1.5 Å h^{-1} to 9 Å h^{-1} (Deposition time adapted to get ≈ 1/3 ML layer thickness)
Substrate temperature	650 K	503 K	300 K to 800 K
Pressure during evaporation	$\leq 5 \times 10^{-10}$ mbar	not given	$\leq 3.5 \times 10^{-10}$ mbar
Base pressure	$\leq 2 \times 10^{-10}$ mbar	not given	$\leq 1 \times 10^{-10}$ mbar

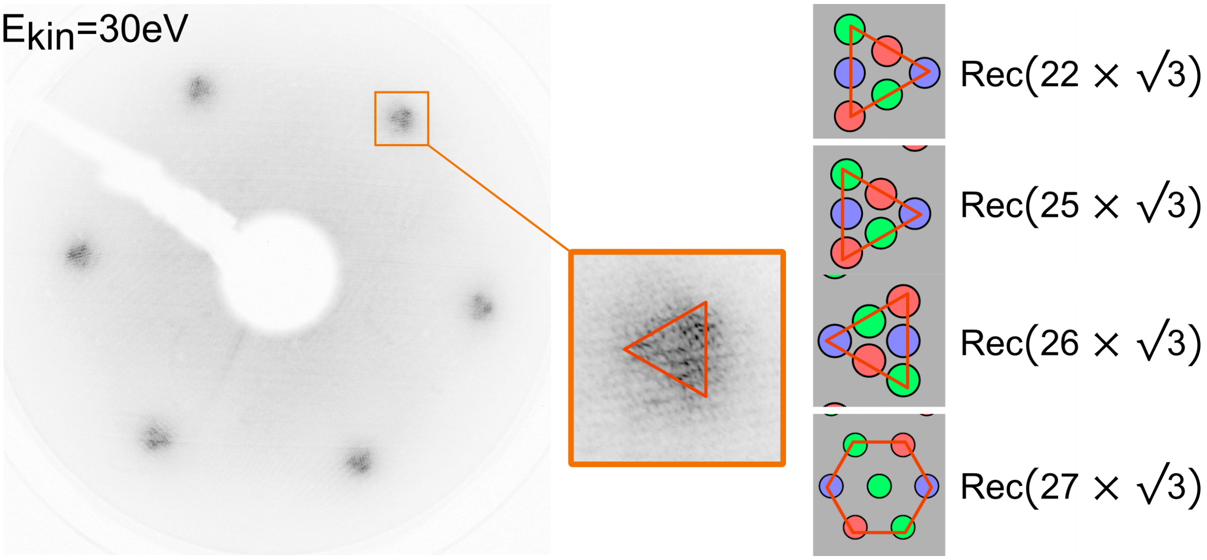

Figure S4. Comparison of calculated LEED pattern (calculated by LEEDPat[2]) for different strained surface reconstructions. Marked by the orange square is a $\sqrt{3}$-spot with its surrounding reflex pattern stemming from the long-range order. Similar to the pattern analysis conducted by Shah et al.[3], it is evidend, as marked by the red triangle, that only the $\mathrm{Rec}(26 \times \sqrt{3})$-periodicity resembles the observed LEED pattern. The $\mathrm{Rec}(22 \times \sqrt{3})$-as well as the $\mathrm{Rec}(25 \times \sqrt{3})$-periodicity are ruled out because their triangle shape points in the wrong direction. The $\mathrm{Rec}(27 \times \sqrt{3})$-periodicity displays a hexagonal spot pattern which does not resembles the observed triangular-shaped pattern.

References

1. Hochhaus, J. A. Lmfitxps. Zenodo, DOI: 10.5281/ZENODO.8181378 (2024).

2. Hermann, K. LEEDpat download package, DOI: 10.17617/3.8AYKWU (2022).

3. Shah, J., Wang, W., Sohail, H. M. & Uhrberg, R. I. G. Atomic and electronic structures of the Au 2 Sn surface alloy on Au(111). *Phys. Rev. B* **104**, 125408, DOI: 10.1103/PhysRevB.104.125408 (2021).

4. Maniraj, M. *et al.* Structure and electronic properties of the (3 × 3) R 30° SnAu2 / Au (111) surface alloy. *Phys. Rev. B* **98**, 205419, DOI: 10.1103/PhysRevB.98.205419 (2018).
